# Designing custom CRISPR libraries for hypothesis-driven drug target discovery

**DOI:** 10.1016/j.csbj.2020.08.009

**Published:** 2020-08-18

**Authors:** Vaishnavi Srinivasan Iyer, Long Jiang, Yunbing Shen, Sanjaykumar V. Boddul, Sudeepta Kumar Panda, Zsolt Kasza, Bernhard Schmierer, Fredrik Wermeling

**Affiliations:** a Center for Molecular Medicine, Division of Rheumatology, Department of Medicine, Solna, Karolinska Institutet and Karolinska University Hospital, Stockholm, Sweden; b School of Physical and Mathematical Sciences, Nanyang Technological University, Singapore; c Structural Genomics Consortium, Department of Medicine, Karolinska University Hospital and Karolinska Institutet, Stockholm, Sweden; d High Throughput Genome Engineering, Department of Medical Biochemistry and Biophysics, Karolinska Institutet, Stockholm, Sweden; e Science for Life Laboratory, Stockholm, Sweden

## Abstract

Over the last decade Clustered Regularly Interspaced Short Palindromic Repeats (CRISPR) has been developed into a potent molecular biology tool used to rapidly modify genes or their expression in a multitude of ways. In parallel, CRISPR-based screening approaches have been developed as powerful discovery platforms for dissecting the genetic basis of cellular behavior, as well as for drug target discovery. CRISPR screens can be designed in numerous ways. Here, we give a brief background to CRISPR screens and discuss the pros and cons of different design approaches, including unbiased genome-wide screens that target all known genes, as well as hypothesis-driven custom screens in which selected subsets of genes are targeted (Fig. 1). We provide several suggestions for how a custom screen can be designed, which could broadly serve as inspiration for any experiment that includes candidate gene selection. Finally, we discuss how results from CRISPR screens could be translated into drug development, as well as future trends we foresee in the rapidly evolving CRISPR screen field.

## 1 CRISPR

Since the first publications where CRISPR/Cas9 was used as a controlled molecular biology tool in 2012–2013 [2], [3], [4], the use of CRISPR systems has found its place as a staple in the researcher’s toolbox. These experimental CRISPR/Cas9 tools are developed from naturally occurring systems found in multiple bacterial and archaeal species [5], [6], [7], [8], [9], [10]. Many developments of the CRISPR system have been presented over the last few years, including the use of Cas9 variants from other bacteria, modified Cas9 with novel functionalities, as well as using other Cas proteins [11], [12], [13], [14], [15]. As of 2020, the CRISPR field is very active, and further exciting developments can be expected.

The basis for a standard CRISPR/Cas9 experiment intended to inactivate a gene is the formation of a complex of a guide RNA (gRNA), and the endonuclease Cas9. As the gRNA/Cas9 complex binds the specific genomic DNA sequence, dictated by the gRNA sequence, a DNA double-strand (dsDNA) break is introduced in the genomic DNA by the activity of Cas9 [2]. Cells have inherent mechanisms for rapidly repairing dsDNA breaks, but these are error-prone and commonly result in small insertions and deletions (indels) of nucleotides at the repaired site [16]. Notably, if the dsDNA break is repaired without indel formation, it is likely recut by the gRNA/Cas9 complex until indel formation will eventually preclude further recognition and recutting. If the gRNA is designed such that the double-strand break is localized in a protein-coding part of a gene, there is a high likelihood that resulting indels cause a frameshift and thus a knock out allele of that gene [17], [18]. This review will not further focus on technical details related to how to perform CRISPR experiments; excellent overviews of this can be found here [19], [20], [21].

### 1.1 CRISPR-based screens

In a screen, various interventions are tested in parallel, and the result on the assayed target is recorded for the different interventions. Screening approaches have historically been extensively used by the pharmaceutical industry to identify chemically synthesized small molecule drugs that affect various cellular phenotypes linked to disease [22]. Typically, such screens are performed in large scale high-throughput formats where each molecule is tested in separated wells, testing a vast number of molecules in parallel to identify those that affect the studied behavior.

In a CRISPR screen, multiple gRNA-based perturbations are introduced into a cell population, and genes that affect the studied phenotype are identified (Fig. 2). The first genome-scale CRISPR screens were published in 2014 [23], [24], [25]. Several significant developments have been presented since, showing the immense potential of CRISPR screening technologies. These potentially transformative advances involve methods using (*i*) single-cell RNA sequencing (scRNAseq) based readouts for CRISPR screens (which has been referred to as Perturb-seq, CRISP-seq and CROP-seq [26], [27], [28], [29], (*ii*) the ongoing construction of CRISPR screen databases identifying genes essential for the survival of various human cancer cells [30], [31], [32], [33], and (*iii*) CRISPR activation (CRISPRa) based screening using a modified Cas9 version that does not inactivate genes, but instead brings *trans*-activating elements to the transcription start site of a gene, thus activating gene expression [34], [35], [36]. CRISPRa share features with the CRISPR interference (CRISPRi) system, where a modified Cas9 version is used that causes transcriptional repression of the targeted gene [34], [37], [38].

**Fig. 1:**
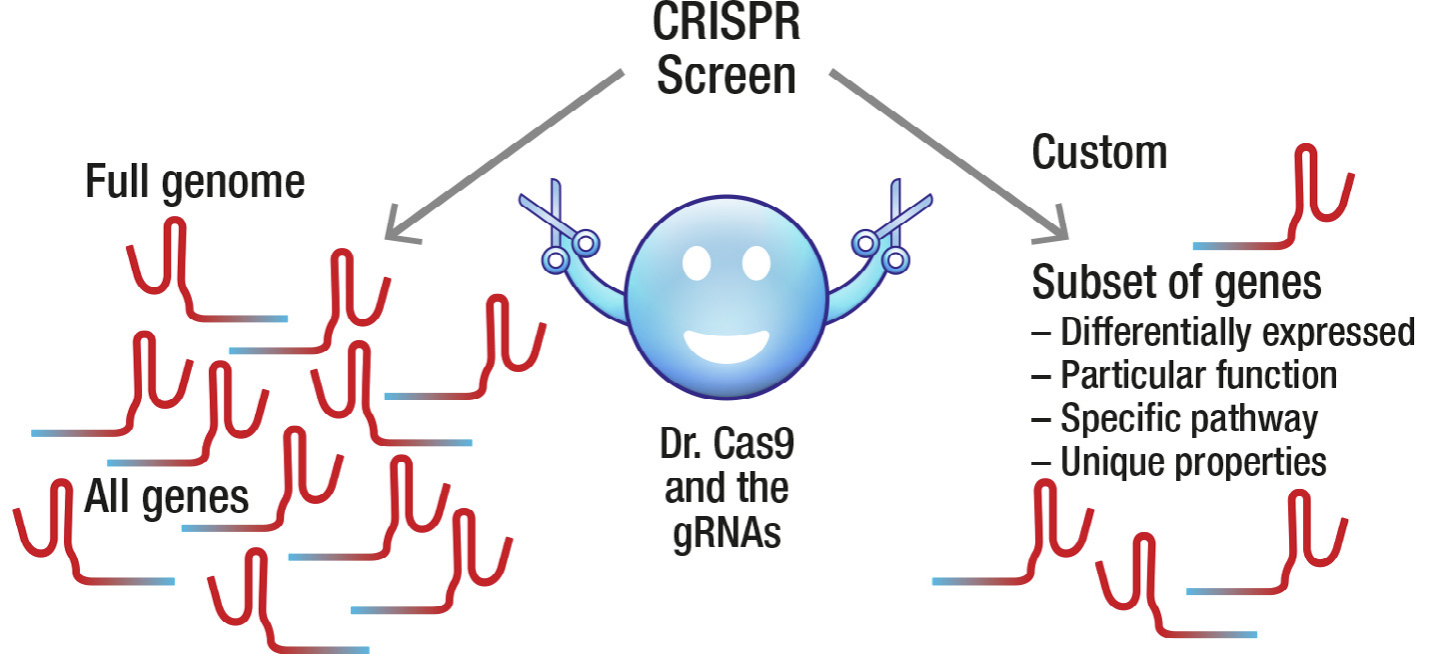
Illustration highlighting the difference between genome-wide and custom screens (1).

**Fig. 2:**
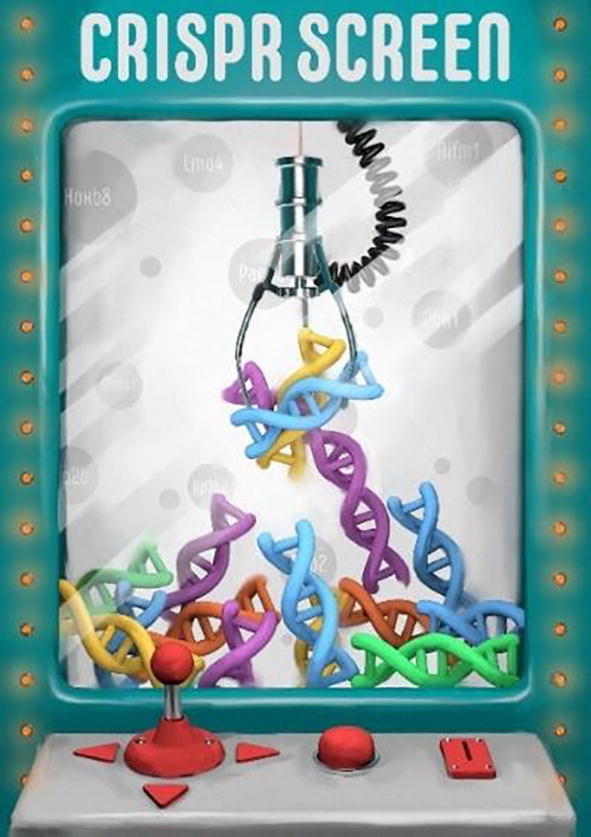
Illustration of a CRISPR screen.

The rapid development of CRISPR screening approaches can be attributed in part to the fact that the generic setup builds on similar, previously established methods. In particular, whole genome shRNA (short-hairpin RNA) based screens share many similarities in the experimental setup and analysis to gRNA (CRISPR) based screens [39], [40], [41], [42]. Based on the different modes of action between shRNA and gRNA, with shRNAs causing degradation of gene transcripts, while gRNAs directly targeting the gene itself, one could expect differences between these approaches. Conceptually, an shRNA approach will give a range of degrees of target inhibition, while a gRNA approach will have a more binary, all or nothing pattern of inhibition on a per cell basis. Systematic comparisons between shRNA and gRNA-based screens have been performed. While gRNA-based screens are typically more effective, the two approaches likely can serve as useful complements to each other in certain situations [43], [44], [45].

**Fig. 3:**
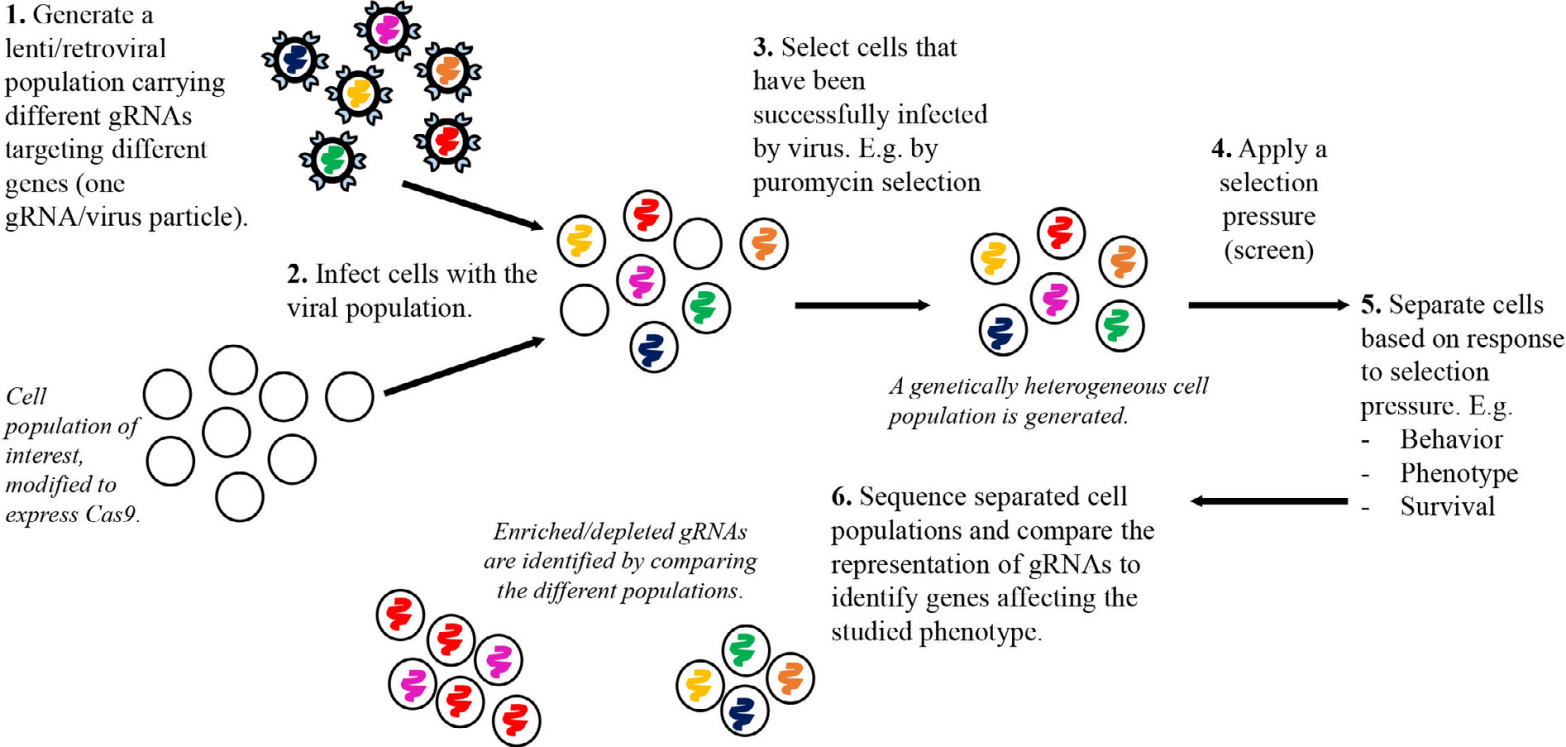
Representation of a pooled CRISPR screen. Lenti- or retroviral particles are typically used for delivery as they can (i) be titrated to achieve a specific infection rate, (ii) will integrate into the genome of the infected cell, and (iii) infect many different cell types. The integration enables simple quantification of the gRNA representation in different cell populations by next-generation sequencing, and the subsequent identification of enriched or depleted gRNAs comparing different populations.

A crucial development for genome-wide shRNA and subsequent CRISPR screens was the development of massively parallel sequencing technologies at the end of the 1990 s that became commercially available around 2005 [46]. This approach enabled performing large scale screens in pooled samples (referred to as *pooled* screens), instead of each perturbation having to be separated into individual wells (which is referred to as *arrayed* screens). In a pooled screen (see Fig. 3), genetic perturbations mediated by, for example, gRNAs are applied to a cell population by retroviral or lentiviral delivery, leveraging the ability of these viruses to be titrated to a concentration at which few cells will be infected by more than one virus particle and thus one gRNA, and that the virus integrate into the genome of the infected cell. After the selection of infected cells, a controlled, genetically heterogeneous cell population is thus generated, where each cell will have one gene targeted by one gRNA construct that is integrated into its genome. Standard massively parallel sequencing of PCR-amplified genomic DNA, using primers amplifying the gRNA constructs, are then used to identify the representation of different gRNAs in the studied cell population. As a readout, the pooled screen could, for example, be used to identify genes that are central for the survival of a cancer cell by comparing the gRNA representation in the cancer cell population directly after infection to a later time point when the cells have been expanding *in vitro* or *in vivo* [47], [48]. As such, gRNAs depleted from the sample taken at the later time point target genes that are essential for the expansion and/or survival of the cancer cell. Open-source software packages used to analyze gRNA representation in sequencing data, including MAGeCK [49] and pipelines such as CRISPRanalyzeR [50], have significantly facilitated the use of CRISPR screens for labs with limited bioinformatics experience.

### 1.2 Which genes should be targeted in a CRISPR screen?

Conceptually, there exist at least three overall approaches related to the genes to be included in any screen. Here we broadly refer to these as genome-wide, restricted, and hypothesis-driven, where the latter two could be categorized as custom or focused approaches.

1. Genome-wide screens; targeting all known genes. This approach is unbiased in its design and thus has the highest potential to identify unexpected genes affecting the studied phenotype. Several genome-wide libraries currently exist and can be obtained at a relatively low cost from, for example, the non-profit plasmid repository Addgene [17], [36], [47], [48], [51], [52], [53], [54], [55], [56], [57], [58], [59], [60]. However, targeting roughly 20,000 genes and controls comes with challenges. Typically, 4–10 gRNAs are recommended to be included for each gene in such screens, which results in at least 80,000 different gRNAs in a genome-wide screen. Furthermore, for proper library representation, it is suggested that at least 500 cells per gRNA should be used, resulting in 40 million cells per experimental group in a pooled screen. In addition, a low multiplicity of infection (MOI; the ratio of viral particles to cells that are being infected) is required to assure that few cells are infected with more than one gRNA each. To achieve this, the viral library is often diluted to a level where 25–30% of the cells are infected [20]. Thus, three to four times more cells than needed for the screen must be initially transduced. In cells that are challenging to infect, achieving a 25–30% infection rate could be difficult, and an even larger starting cell population might be required. Of course, the number of cells needed is further increased by biological replicates and different interventions included in the screen design.

Minimum number of cells needed for a genome-wide CRISPR screen:

20 000 targets × 4 gRNAs/target = ~80 000 gRNAs

80 000 gRNAs × 500 cells/gRNA = ~40 million cells per group

(25% infection rate = need to start with greater than 160 million cells)

A full genome CRISPR screen thus requires a large number of cells, which could pose a problem for the specific experiment. Established cancer cell lines are typically easy to expand to unlimited numbers. In contrast, specific primary cell populations and cells from patient material can be difficult or impossible to obtain in sufficient numbers. The specific screening setup can also dictate other limitations. For example, *in vivo* mouse cancer experiments have limitations for the number of cancer cells that can be administered to the recipient mouse. A standard experimental protocol for subcutaneous grafting of cancer cells, subsequently studying the *in vivo* growth profile over time, would typically involve the administration of fewer than one million cells, which is far from the 40 million cells needed for a genome-wide screen in the administered cancer cells.

One approach that could facilitate screening in situations where the number of cells is limited is to include unique molecular identifiers (UMIs) in the CRISPR library [61], [62]. This has been shown to increase the power of the analysis by combining information about gRNA representation with the clonality of the cells, adding another layer to the analysis. Such an approach could make it feasible to screen fewer cells per gRNA with retained statistical power.

2. Restricted screens; focusing on subsets of genes that for different reasons seem relevant. For example, this could be all kinases, all transcription factors, all G-protein coupled receptors etc. This review will not further discuss such approaches in-depth. However, these approaches could be a plausible start for a screen where the total number of genes that can be targeted is limited, but where a clear hypothesis as to the regulation of a studied phenotype is lacking. Several restricted libraries are available through Addgene and commercial providers. Multiple resources for identifying subsets of genes can also be used to design restricted libraries de novo. These resources include the *Human Protein Atlas*, comprising a compiled database for easy identification of categories of genes to be included [63]. The *Gene Ontology (GO)* database is another useful resource to identify smaller and larger subsets of genes, categorized as GO-terms [64].

3. Hypothesis-driven, custom screens; targeting genes experimentally identified to potentially be involved in the studied behavior. These types of screens could start with a list of genes generated by, for example, an OMICS experiment. Many different approaches exist to generate such candidate lists of genes that potentially affect a phenotype of interest. These include RNA-based methods such as RNAseq, single-cell RNAseq [65], and spatial transcriptomics approaches [66], DNA-based methods such as ChIP-seq [67] and ATAC-seq [68], as well as protein-based approaches like mass spectrometry, phosphoproteomics [69], and different affinity and proximity-based methods like BioID [70]. The candidate gene list could also be derived from previous larger CRISPR screens or from in silico predictions.

The rest of this review will primarily focus on such hypothesis-driven screens. Here the concept is to use a CRISPR screening approach to identify which of the genes, identified by e.g. RNAseq, that are central to the studied cellular behavior, aiming to extract functional information from a descriptive OMICS data set. The overall aim of the study could, for instance, be to describe the foundations of a cellular phenotype and to identify potential drug targets that could be targeted to modify the phenotype. A custom hypothesis-driven screen could, in a simple form, target all significantly up and downregulated genes in the studied cell population with the hypothesis that differentially expressed genes are likely involved in the studied phenotype. Initial transcriptomics data could be complemented by including, for example, proteomic data, as many proteins are not primarily regulated on the transcriptional level, but on a post-transcriptional level. Independently of which method, or combination of methods, that is used to generate the candidate gene list, it is likely not going to contain all critical genes of the studied system. Different approaches to link additional genes to the initial OMICs generated candidate list will be discussed in the following section.

A custom screen design can enable discovery in cases like the *in vivo* mouse cancer model highlighted above, where a genome-wide CRISPR screen would not be feasible. Such an experiment could, for example, be aimed at identifying genes affecting how cancer cells survive as a drug is administered to the mouse. In a study with a similar setup, Manguso et al. elegantly solved the problem related to the limited amount of cells that could be administered to a mouse, by only including genes in the screen that (*i*) belonged to specific relevant categories of genes (kinase, phosphatase, cell surface, plasma membrane, antigen processing and presentation, immune system process, and chromatin remodeling based on GO-terms), and (*ii*) had an expression level above a pre-determined threshold [71]. Limiting the screen in such a way allowed the screen to include only around 10% of the number of genes needed in comparison to a genome-wide screen. Manguso et al. furthermore divided the gRNA library into four sub-libraries with different gRNAs, where each library contained gRNAs for all genes, but only one gRNA per gene. The four different sub-libraries were then used in parallel mouse experiments, and the results from the four different screens were combined to generate data that thus included four gRNAs per gene; in the end, generating impressive resolution of the screen despite the limitations of the assay.

In summary, several parameters need to be considered when deciding how many genes to target in a screen. Genome-wide screens have the highest possibility for unbiased discovery, although a large number of cells need to be included, which can pose a significant challenge. More targeted, restricted or hypothesis-driven screens are more straightforward, but can only identify genes that are included in the screen library. Genome-wide and different types of restricted libraries are readily accessible, whereas custom libraries need to be synthesized and cloned into the CRISPR plasmid of choice. This is more labor-intensive and typically more expensive than buying readymade libraries. However, the cost of generating custom libraries is typically not prohibitively high.

## 2 How to design a hypothesis-driven custom screen

In a hypothesis-driven custom CRISPR screen, we suggest starting with a list of genes experimentally identified in the studied system. Differentially expressed genes identified by RNAseq comparing a tumor treated with a drug or control is an example to which we return. Here, the hypothesis would be that the differentially regulated genes are involved in the biological activity of the drug and that a screen could identify which of these genes are central to the phenotype.

In the following section, a number of different analytical tools that can be used to expand the list of genes to include in a custom screen, are introduced. This is not a comprehensive list but focuses on simple and freely available tools we find helpful. Table 1 below provides more information and links to the suggested resources.

**Table 1:** Online tools and databases.

Tool	Location	Comment
MAGeCK	https://sourceforge.net/p/mageck/wiki/Home/	An open source computational tool for CRISPR screen analysis.
CRISPRanalyzeR	http://crispr-analyzer.dkfz.de	Web based tool for CRISPR screen analysis.
Addgene	https://www.addgene.org/crispr/	A non-profit plasmid repository where many CRISPR relevant plasmids, and pooled gRNA libraries can be obtained.
The Human Protein Atlas	https://www.proteinatlas.org/humanproteome/proteinclasses	Database including protein class categorization (including drug targets), as well as extensive expression information from human cells and tissues.
Gene Ontology (GO) database	http://geneontology.org/	Extensive categorization of genes into GO-terms.
Gene Set Enrichment Analysis (GSEA)	https://www.gsea-msigdb.org/gsea/index.jsp	Pathway analysis tool for RNAseq data.
g:GOSt of g:Profiler	https://biit.cs.ut.ee/gprofiler/gost	Pathway analysis tool based on lists of manually input genes.
Mouse Genome Informatics (MGI) Gene Ontology Browser	http://www.informatics.jax.org/vocab/gene_ontology/	Simple tool to search GO-terms.
g:Converter of g:Profiler	https://biit.cs.ut.ee/gprofiler/convert	Tool that can be used to convert Gene Ids, and to extract genes from GO-terms, KEGG pathways etc.
Pathway Commons	https://www.pathwaycommons.org/	Analyses lists of genes and shows interactions and enriched pathways.
GeneMANIA	https://genemania.org/	Analyses lists of genes and shows interactions and enriched pathways (plugin for Cytoscape also exists).
Harmonizome	https://amp.pharm.mssm.edu/Harmonizome/	Database that extracts information from multiple other sources and integrates it into a search feature
Geneshot	https://amp.pharm.mssm.edu/geneshot/	Literature mining tools providing lists of genes linked to the search term(s).
Green Listed tool	http://greenlisted.cmm.ki.se/	Rapid gRNA design tool for custom CRISPR screens. Can also be used to extract non-targeting and intergenic control gRNAs (select Zhang/GeCKOv2 or Wang/Lander/Sabatini and press “Detailed Information”).
Depmap portal	https://depmap.org/portal	Cancer dependencies analytical and visualization tools, which e.g. can be used to identify essential genes.
g:Orth of g:Profiler	https://biit.cs.ut.ee/gprofiler/orth	Translates gene identifiers between organisms.
MGI batch query	http://www.informatics.jax.org/batch	Tool that can identify alternative names of genes.
Drug Gene Interaction	http://www.dgidb.org/druggable_gene_categories	Database of drug targets.
Probe Miner	https://probeminer.icr.ac.uk/#/	Database of small molecule drugs and their targets.

### 2.1 Identifying genes linked to your data set

A common starting point for evaluating an OMICs dataset is a pathway analysis, and several tools exist to perform such. Genes identified by the pathway analysis could be included in the subsequent screen library, complementing the original OMICs dataset. In such a way, the screen is expanded by thoroughly exploring the involvement of pathways linked to the original data set. An excellent recent protocol by Reimand et al. describes some approaches that can be used to perform pathway analysis [72].

One of the most advanced freely available analysis tools for pathway analysis of RNAseq data is the *Gene Set Enrichment Analysis (GSEA)* [73]. In the standard setup, GSEA takes the expression levels of all genes in the expression data set and compares them to defined gene sets to identify pathways linked to the expression profile.

Another useful pathway analysis tool is *g:GOSt* of *g:Profiler* [74]. In contrast to GSEA, g:GOSs is not restricted to uploading a complete expression data set. Instead, the researcher enters a list of genes, and the tool identifies pathways (e.g. GO, KEGG, Reactome and WikiPathways) in which the entered genes are enriched. Additionally, information on transcription factors and microRNAs that could be involved in regulating the gene set can be obtained. Importantly, in contrast to GSEA, the design of this tool allows for the analysis of lists of genes identified based on methods beyond RNA expression.

The *Gene Ontology (GO)* consortium [75] is one of the most popular resources for classifying genes into categories. The tool performs GO-term enrichment analysis and extracts lists of genes linked to the identified GO-terms (e.g. “neutrophil migration” = GO:1990266, which includes 118 genes). The Mouse Genome Informatics (MGI) Gene Ontology Browser is another simple tool for browsing mouse GO-terms. *g:Converter* is an additional convenient tool to extract lists of genes linked to different GO-terms, but also from e.g. KEGG, Reactome, and WikiPathways.

*Pathway commons* is a web-based tool that analyzes a gene set integrating data related to e.g. biological pathways and physical interactions from various publicly available databases [76] such as Reactome, and PANTHER. Pathway commons allows the user to identify related pathways and suggests potential interactions between genes in the entered gene set.

**Fig. 4:**
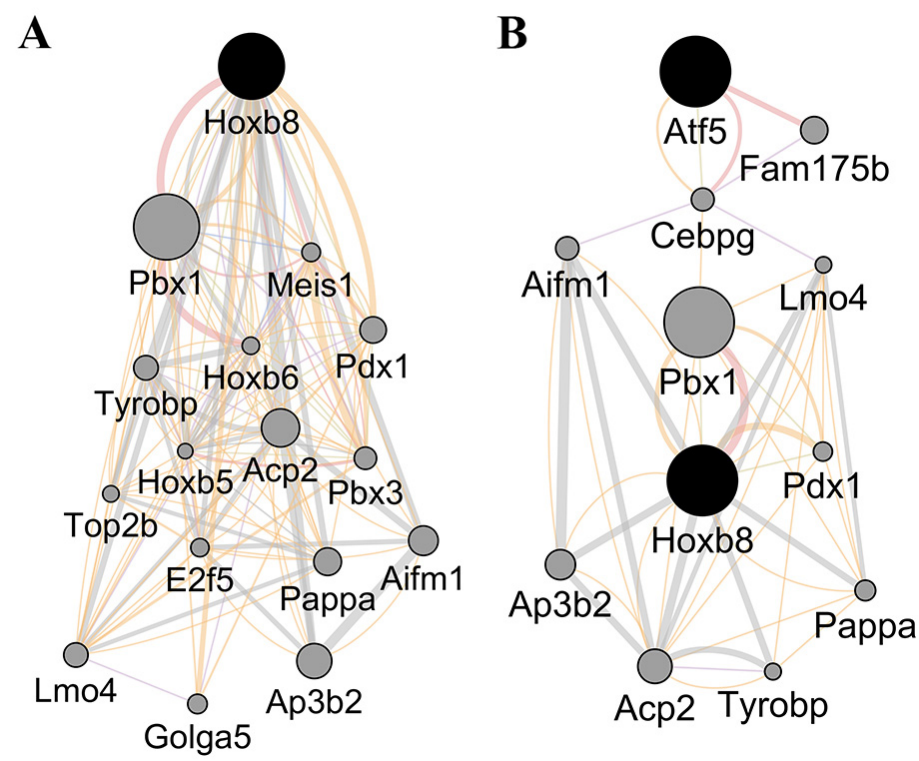
Analysis using the geneMANIA plugin for Cytoscape. (A) Genes identified to be linked to HoxB8. (B). Genes identified to connect Atf5 and HoxB8.

A very useful tool that shares functionalities with Pathway Commons is *GeneMANIA* [77]. A plugin version for Cytoscape of GeneMANIA [78] also exists and is the primary tool we currently use to identify genes to include in our custom screens. The GeneMANIA plugin can quickly analyze large lists of genes and generate graphical representations of gene interactions. Importantly, GeneMANIA can also suggest genes that are linked to a gene or a list of genes. The tool links genes based on factors such as physical interactions, co-localization, and co-expression. It is also possible to define which organism the data should be related to. Fig. 4A shows the graphical output of GeneMANIA when the Hoxb8 gene was entered, including 15 linked genes suggested by the software. The size of the grey circles (nodes) represents link strength, and the color of the connecting lines (edges) represents different categories of interactions.

GeneMANIA can also identify suggested connections between two, or more, genes that are not directly interacting, as exemplified in Fig. 4B by searching Hoxb8 and Atf5, where Cebpg, Lmo4, Aifm1, and Pbx1 are suggested as connecting nodes.

*Harmonizome* [79] is yet another impressive example of a database extracting information from multiple sources. This tool can, for example, identify transcription factor binding to the promoter of an entered gene, pathways the gene is involved in, as well as the expression level and importance of the selected gene for the survival of different cell lines.

Finally, *Geneshot* [80] is a literature mining tool identifying genes often mentioned in context with the query gene in the literature.

### 2.2 Control genes

Another vital part of designing a custom screen is to include positive and negative control gRNAs. The purpose of these are to establish a baseline for the screen, defined by the negative controls, and for evaluation of the efficiency of the screen, shown by the strength of the enrichment/depletion of positive controls. Including 5–10% of the total gRNAs as controls in a screen is a suggested setup. However, in smaller, hypothesis-driven screens, including a higher proportion of negative controls should be considered. The readout of a CRISPR screen is enrichment and depletion of gRNAs comparing experimental groups. In a genome-wide screen, many of the included genes will not affect the studied phenotype, and as a consequence, gRNAs targeting these genes will act as a baseline facilitating the identification of enriched/depleted gRNAs. In contrast, a smaller hypothesis-driven screen would likely be designed to have a higher proportion of the included genes that affect the phenotype, and fewer that act as a baseline. As a consequence, the enrichment/depletion of gRNAs might not be as noticeable depending on the readout used. Therefore, in very small screens, the use of up to 50% negative controls could be considered.

#### 2.2.1 Negative controls

Negative controls are gRNAs that are expected to not affect the studied phenotype. Two types of negative controls are typically considered; (*i*) gRNAs that do not bind anywhere in the studied genome, so-called non-targeting controls (NTCs), and (*ii*) gRNAs that bind intergenic regions that are predicted to not affect any protein-coding gene. The rationale for using “intergenic controls” is that they do not affect any known gene, but still control for the DNA damage response that is triggered when the gRNA/Cas9 complex cuts the genome, which is something the NTC does not control for. In this context, it is worth noting that the DNA damage response induced in a CRISPR experiment can have a negative impact on cell survival and proliferation, thereby introducing noise in a screen [81], [82]. Importantly, a gRNA that binds to multiple genomic regions, having a high off-target activity, will cause an increased DNA damage and non-specific toxicity to the cell [83]. Combining NTC and intergenic controls can be useful if possible. The negative control population should theoretically neither be enriched nor depleted when comparing experimental groups. In practice, NTC sets are commonly slightly enriched, due to the absence of DNA damage induced by the NTC in contrast to targeting gRNAs. A resource to extract lists of negative control gRNAs for mouse and human can be found using the ‘Green Listed’ tool [84]. Pressing “detailed information” in the Zhang/GeCKOv2 or Wang/Lander/Sabatini reference libraries provides more information. Intergenic controls targeting well-defined loci like AAVS1 could also be considered [4].

#### 2.2.2 Positive controls

Positive controls that are known or expected to affect the studied phenotype should always be included, if available. Typically, essential genes that affect cell survival could be considered standard positive controls. As such, gRNAs targeting essential genes are expected to be depleted from the cell population over time. Examples of lists of essential genes can be found in e.g. Hart et al. (Supplemental Table 2 in [58]). Another useful resource for identifying essential genes is the *Depmap portal*, where e.g. result from the Project Achilles can be accessed [85], containing genome-wide gene essentiality data for more than 600 human cell lines. For other screening readouts, such as those based on FACS sorting to isolate cells with different phenotypes, the inclusion of gRNAs against genes coding for the proteins that the FACS antibodies bind to is advisable as positive controls. These gRNAs are expected to be depleted from the sorted population. Similarly, in screens studying the response to a ligand, including a gRNA targeting known receptors for the ligand is a good strategy, as is including gRNAs against GFP in screens using GFP reporters as a readout.

### 2.3 Converting gene identifiers

A common challenge in handling OMICS data are inconsistencies in gene identifiers, either within a species or between different species. There is no simple general approach to solve this problem. We typically use two websites to identify alternative names: The *g:Convert Gene ID conversion* tool or the *g:Orth Orthology search* of g:Profiler, as well as the *Batch Query Tool* of the MGI webpage. As a last resort, alternative names need to be searched manually.

### 2.4 Generating lists of gRNAs from a list of genes

After generating a list of genes and controls to include in a custom screen, we typically use the free web-based ‘Green Listed’ tool for rapid gRNA design, something we have successfully used for several published and unpublished custom screens [84], [86]. Extensive information about how to work with the tool can be found on the webpage, as well as in [1]. Briefly, a Reference Library is selected, a list of genes to be targeted is entered, and adapter sequences for downstream cloning are provided by the user, and Green Listed produces a list of gRNAs to order.

## 3 Trends and future development in CRISPR-based screening

The CRISPR field is rapidly changing, and significant developments can be expected. Here follow some concepts we anticipate will be further developed going forward.

### 3.1 Using different CRISPR systems for screening

Multiple diverse CRISPR systems have been used for genome-wide screening, including systems based on nuclease deactivated Cas9 (dCas9) [34], [35], [36]. Still, there are additional CRISPR-based systems that have features that make them potentially attractive from a large scale screening point-of-view, for example, base editors [87], Cas13 [88] and different epigenetic modifying dCas9 versions [89]. A bottleneck establishing novel CRISPR-based screening platforms is the development of reliable gRNA prediction tools for the specific setup, allowing for large scale design of gRNAs without the need to validate individual gRNAs [17], [18], [90]. Thus, reliable gRNA prediction algorithms need to be developed for the specific system to enable genome-wide screens.

Alternative gRNA design approaches could potentially be considered, for example, where gRNA sequences are generated from mRNA or DNA isolated from the cells to be screened [91], [92]. Such approaches would, however, likely demand a significantly larger amount of cells to be included in the screen as the activity of the generated gRNAs as a population must be expected to have low activity.

### 3.2 Screens identifying pathways involving genes with overlapping functionality

A standard CRISPR screen setup, where each cell is targeted by one gRNA, has a good chance of identifying genes that have a non-redundant activity in the studied behavior. However, if redundancies exist in an involved pathway, for example, if several genes have overlapping functionality, these genes will typically not be identified in the screen. For drug target discovery, this does not pose a significant problem, as a drug target optimally should have a non-redundant functionality in the pathway the drug is intended to interfere with. Still, from a biological point of view, identifying pathways involved in a studied behavior is an important development, even if functionally overlapping genes are involved. Several approaches could be considered going forward with a project focused on detailed understanding of the involved biology. For example, performing the screen in cells that have been generated to lack genes thought to contribute to redundancies in a pathway of interest. Another approach is screening with combinations of more than one gRNA in each cell. Such screens have been elegantly performed by using orthogonal Cas9 version [93], [94]. Importantly, controlled combinatorial screens rapidly become prohibitively large as the number of included genes increase. For feasibility, such screen approaches need to be more limited, targeting subsets of genes. Finally, performing screens based on both inactivation of genes (such as traditional Cas9-based screens or CRISPRi) with screens based on activation of gene expression, CRISPRa, could significantly increase the biological understanding of a system. Taken together, we foresee more intricate CRISPR screen designs allowing for dissecting of complex biological pathways [93].

### 3.3 The DNA damage response challenge

Standard CRISPR experiments, where dsDNA breaks are formed by the gRNA/Cas9 complex, results in a DNA damage response in cells, which introduce non-specific noise into screens [81], [82], [95], [96]. The type of cells used will likely influence this problem, for example, related to the specific status of TP53 in the cell, central to the DNA-damage response. Different approaches to limit the negative impact of the DNA damage response will likely be an integrated part of future CRISPR screen projects, for example, by performing screens in TP53 KO cells, by applying transient TP53 inactivation, or by using CRISPR versions that do not induce a DNA damage response, such as CRISPRa- and CRISPRi-based screens.

### 3.4 Performing CRISPR screens in more complex biological settings

Large scale screening efforts have identified common and specific patterns related to the survival of cancer cell lines cultured in well-defined *in vitro* conditions [32], [97], [98]. Importantly, the result of a screen will be linked to the selection pressure applied by the screen setup. Therefore, further discoveries can likely be obtained by introducing layers of relevant complexity in the screen design. For example, performing *in vivo* screens in the context of animal disease models, or in more complex co-culture settings, including different organoid designs, have the potential to identify prospective drug targets that are not found in a standard *in vitro* survival screen setup [71], [99], [100], [101], [102], [103]. Performing screens with primary patient material, where the unique genetic and epigenetic state of the patient contributes to the complexity, could also be highly relevant for discovery. A somewhat similar but potentially more feasible setup is to generate cell lines with patient-specific mutations and perform screens in these, aiming to identify how the specific mutation is affecting the cellular behavior.

All of these more complex screen settings are limited by multiple factors, including the number of cells that can successfully be infected with the CRISPR library, how long the studied cells survive, the possibility of developing relevant readouts that the cells are separated based on in the screen, etc. We argue that using custom CRISPR screen approaches, as discussed above, could be a powerful way to approach these more complex screen setups and still retain discovery potential.

### 3.5 Translating results from screens into drug development

The identification of candidate gene and protein targets linked to disease often serves as an initial step in contemporary drug development. In this regard, a CRISPR screen can identify genes that are central to a studied pathway, for example related to T cell activation [104]. Conceptually, the phenotype resulting from efficient targeting of a gene with an inhibitory drug should recapitulate, at least in part, the phenotype, as a result of a knockout of the same gene. A CRISPR screen could thus serve as a rapid platform to prioritize drug target candidates.

However, only 5–10% of the protein-coding genes are considered to be “druggable” with small molecule drugs. This is based on structural characteristics, where druggable proteins have a three-dimensional structure that allows for a small molecule to dock into a unique pocket and thereby affect the function of the protein. Commonly, such a pocket is the active site of an enzyme. Prediction algorithms have suggested genes referred to as the druggable genome [105], [106], [107]. Lists of validated and potential drug targets can be found, for instance, through the *Human Protein Atlas* [63] and the *Drug Gene Interaction Database* [108]. Already developed small molecule drugs targeting different proteins can also be found, for example, through the ‘*Probe Miner*’ tool [109].

The development of biological drugs, like antibodies and soluble receptors, adds another mode of action to affect cellular behavior [110], [111]. Secreted proteins and proteins exposed on the cell surface are apparent candidates for biological drugs. Nevertheless, the observation that current drug modalities cannot target most human proteins still holds true also when including biological drugs, and translating results from a CRISPR screen to drug development is therefore not necessarily straight forward.

#### 3.5.1 RNA interference and antisense oligonucleotides

A less explored therapeutic approach, which is not limited by specific features of the target’s protein structure, is based on RNA interference (RNAi) and antisense oligonucleotide (ASO) technologies that can be designed to inhibit any coding or noncoding RNA [112], [113].

RNAi is based on different small RNA oligonucleotides, including shRNAs mentioned above, that are complementary in sequence to their target RNA, thus forming the basis of their specificity and ability to target the specific RNA. The RNAi molecules interfere with their target RNA and cause its degradation through the activity of the RNA-induced silencing complex (RISC) [114], [115]. ASOs, on the other hand, are composed of a DNA-based oligonucleotide that comprises a chemically modified backbone and modified bases that aid in stability and activity [116]. Similar to RNAi, ASOs are designed to be complementary to the target RNA sequence. The binding of the ASO to its target RNA results in an ASO-RNA complex that recruits the RNAse H enzyme and the subsequent degradation of the RNA [117].

RNAi and ASO-based drugs could thus be designed to target any gene identified in a CRISPR screen, independently of whether the target is "druggable" or not. Nonetheless, there are significant challenges to these types of drugs, including the difficulty of delivering the drug to the correct cell and the potentially higher cost compared to traditional small molecule drugs. Different approaches have been explored to solve these challenges e.g. using lipid carriers aiming for more specific delivery to the cells of interest [118], [119]. The carriers can be further functionalized by, for example, introducing ligands for specific endocytic receptors, facilitating uptake into cells of interest [120], [121], [122], [123]. Another possible approach is to deliver the RNAi/ASO molecules specifically to the site where the action is intended. For example, in the case of rheumatoid arthritis, RNAi/ASOs could be injected directly into the affected joint as a therapy [124], [125]. Similar concepts could be considered in other contexts where features of the disease are localized, for instance delivery into the cerebrospinal fluid of patients with multiple sclerosis or neurodegenerative diseases, as well as delivery into a tumor or draining lymph node of a cancer patient to trigger a stronger immune cell activation against the cancer.

#### 3.5.2 Using CRISPR as a drug

Yet another potential therapeutic alternative could be delivering CRISPR constructs directly into the patient to modify genes identified to be linked to a disease [126]. However, several complicating factors exist, including the fact that CRISPR systems are derived from prokaryotes, which trigger the activation of the immune system [127], [128]. Delivery of the CRISPR constructs to the correct cells or tissues is also a significant challenge. One alternative approach to administering the CRISPR construct directly into a patient is to extract relevant cells from the patient, modify them *ex vivo*, and reintroduce them back to the patient. Such approaches are currently in clinical trials, both aiming to correct inherited genetic modifications affecting hematopoietic stem cells that result in severe disease related to the hematopoietic system [129], as well as to generate aggressive tumor-targeting T cells that can be given to cancer patients [130].

**Fig. 5:**
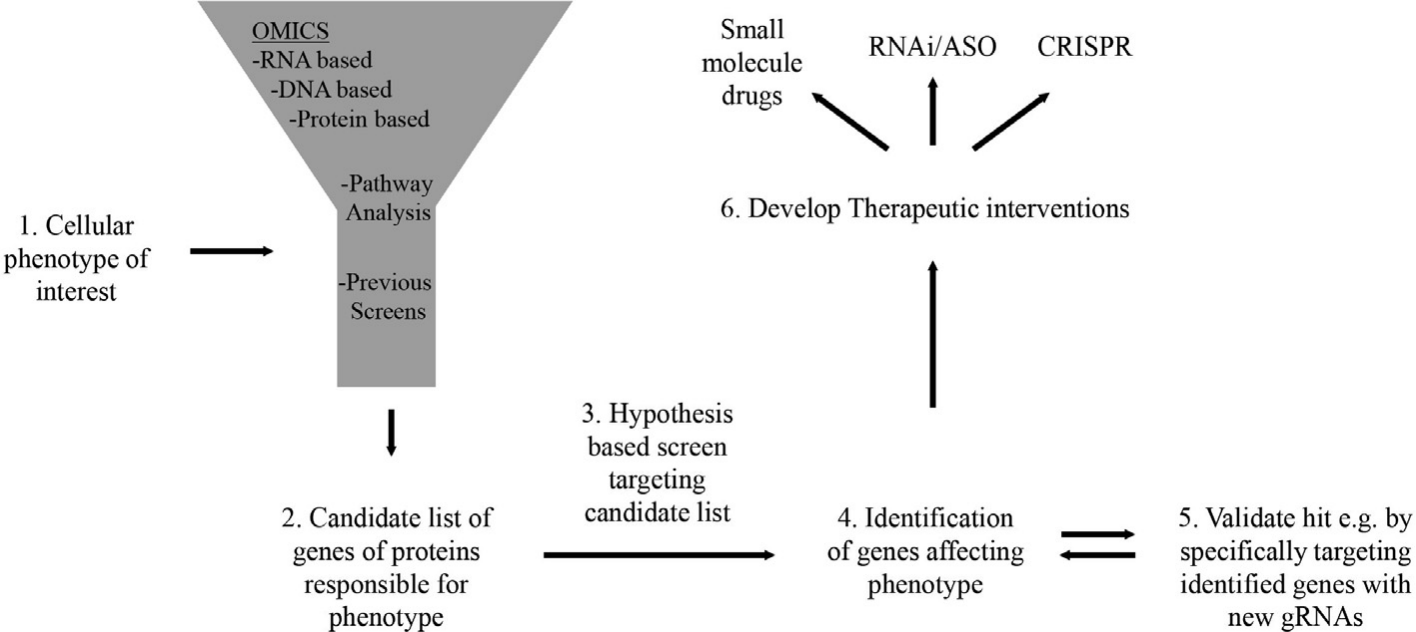
Summary of proposed discovery process.

In summary, over the last decade, CRISPR and CRISPR-based screens have been developed into powerful discovery tools used by the research community. Designing a screen can be done in several ways, where the number of genes included in the screen should be considered. The more genes that are included, the more unbiased result can be expected, but as a consequence, more cells need to be used, which can become a significant technical bottleneck for the screen. We propose using hypothesis-based, custom screens as a rational and straightforward alternative approach and have, in detail, discussed concepts to design such screens using, for example, an RNAseq experiment as a starting point. In this scenario, we aim to transform a descriptive expression dataset into biological understanding. As these smaller screens are much less demanding than genome-wide screens, performing a series of custom screens where the hypothesis is refined for each screen can be a practical discovery approach. Finally, using CRISPR to identify genes that affect a cellular behavior linked to disease can serve as a starting point for drug development. However, since only a limited number of proteins/genes can be targeted with traditional drugs, novel approaches likely need to be explored to develop drugs targeting identified genes. Combining CRISPR screening approaches for discovery with the development of RNAi or ASO based drugs could serve as a foundation for future precision medicine (Fig. 5).

## Declaration of Competing Interest

The authors declare that they have no known competing financial interests or personal relationships that could have appeared to influence the work reported in this paper.
